# Resilience processes among Ukrainian youth preparing to build resilience with peers during the Ukraine-Russia war

**DOI:** 10.3389/fpsyg.2024.1331886

**Published:** 2024-02-20

**Authors:** Francesca Giordano, Shannon Lipscomb, Philip Jefferies, Kyong-Ah Kwon, Marianna Giammarchi

**Affiliations:** ^1^Resilience Research Unit, C.R.I.d.e.e., Department of Psychology, Università Cattolica del Sacro Cuore, Milan, Italy; ^2^Human Development and Family Sciences, College of Health, Oregon State University—Cascades, Bend, OR, United States; ^3^Resilience Research Centre, Faculty of Health, Dalhousie University, Halifax, NS, Canada; ^4^Instructional Leadership and Academic Curriculum, Jeannine Rainbolt College of Education, University of Oklahoma, Tulsa, OK, United States; ^5^Resilience Research Unit, Department of Psychology, Università Cattolica del Sacro Cuore, Milan, Italy

**Keywords:** resilience, war, youth, Ukraine, protective processes, intervention, context

## Abstract

The war in Ukraine significantly impacts the mental health and well-being of its youth. Like other communities affected by war, Ukraine’s youth are at risk of developing psychopathological symptoms, and there is a shortage of mental health and psychosocial support services to address this. Resilience-building initiatives present an alternative approach to supporting the well-being of young people by promoting protective processes to enhance the likelihood of positive development in the context of adversity. Emerging research findings suggest that young people themselves can serve as powerful facilitators of such initiatives with one another. Yet, evidence about culturally and contextually relevant protective processes is needed to guide such interventions, especially among young people experiencing the war and working to boost resilience within their communities. In this study, we identified key protective processes Ukrainian youth depend on as they adapt to the conflict while also preparing to implement a resilience-building intervention as a facilitator. Through thematic analysis of transcripts of three training sessions with Ukrainian youth (*n* = 15, 100% female; aged 18–22), we identified the following themes: positive thinking, sense of control, emotion awareness and regulation, close personal relationships, and community support. Findings also highlighted the cultural and contextual nuance of these protective processes, as well as individual differences in the ways they co-occurred and manifested within each youth. Results have implications for developing tailored yet flexible resilience-building interventions that can be delivered by lay people, including youth with their peers, in Ukraine and other cultures and contexts.

## Introduction

The war in Ukraine has had enormous mental health consequences for its citizens, particularly its youth (Hodes, 2023). A recent study found that 59% of Ukrainians had developed a mental health problem related to the 2022 Russian-Ukrainian war (Chudzicka-Czupała et al., 2023). The impact of war on mental health is widely acknowledged; it is common to encounter increased levels of depressive, anxiety, dissociative, and post-traumatic stress disorder symptoms in affected individuals (Betancourt et al., 2010; Dimitry, 2012; Nyarko and Punamäki, 2021). During these conflicts, young people are often exposed to various war-related disruptions, such as loss of social support, forced relocation, and sometimes abuse and violence, as well as the death of family and friends (Chaaya et al., 2022; Osokina et al., 2023). These experiences have been documented in Ukraine and linked to a significant increase in mental health issues in young Ukrainian people (Osokina et al., 2023).

The psychological literature describes a range of mental sequelae associated with youth’s exposure to war (Werner, 2012). Indeed, the war situation often separates youth from their families, peers, schools, and communities that provide structure, consistency, and support, disrupts their relationships with significant others and hampers developmental processes (Zuilkowski and Betancourt, 2014). It is particularly challenging for youth to cope with traumatic experiences, such as danger, death, violence, and displacement, and in the absence of family or peers, which may result in mental health problems. Research shows that exposure to war increases the risk for mental health issues, with prevalence ranging from 22% (Afghani youth; Panter-Brick et al., 2009) to 97% (former child soldiers in Northern Uganda; Derluyn et al., 2004). Thus, there is an urgent need to understand Ukrainian youths’ experiences during the war and protective processes to reduce their mental health issues and promote their resilience. The current study documents these protective processes specifically among Ukrainian youth who are nurturing resilience both within themselves and with other youth in their communities. The study aims to identify resilience processes and uncover lessons learned for implementing contextually based interventions to nurture resilience in youth experiencing profound community adversities such as war.

### Resilience

Exposure to conflict settings is not always determinative of long-term mental health issues, and youths’ responses to trauma can vary and be malleable (Zuilkowski and Betancourt, 2014). Many youth survive wars and develop into healthy, well-adapted adults despite the adversity and challenges they face (Luthar and Goldstein, 2004). The process of positive adaptation in the context of adversity, resulting in positive outcomes despite such challenges is defined as resilience (Ungar, 2011; Masten, 2018).

Resilience theory provides a framework for investigating and understanding processes of adaptation to adversity and informs the development of preventive interventions (Giordano et al., 2019a, 2019b). For instance, while some older definitions of resilience characterized it as a psychological trait (see Leys et al., 2020), advances in theory and research have resulted in a conceptualization of resilience as a dynamic developmental process that involves drawing on both internal and external resources at different systemic levels (e.g., individual, relational, community/cultural) (Masten, 2018; Masten and Barnes, 2018; Ungar, 2021). Therefore, identifying these resources and bolstering them maximizes the likelihood of youth achieving positive outcomes (Giordano and Ferrari, 2018).

Studies show that negative impacts of adversity in youth can be buffered or reduced by individual protective processes, (i.e., assets), including problem-solving and self-regulation, agency and self-efficacy, acculturation skills, hope, personality, and neurobiological protections (Masten and Narayan, 2012; Giordano et al., 2020). Negative impacts can also be mitigated by various external protective processes, (i.e., resources; Zimmerman, 2013) including strong family ties, attachment relationships, peer relationships, and school climate (Masten and Narayan, 2012; Hall et al., 2014; Peltonen et al., 2014; Zuilkowski and Betancourt, 2014; Veronese et al., 2021).

However, because resilience processes manifest differently across contexts and cultures (Ungar et al., 2007; Meng et al., 2018; Masten, 2021) it is critical that research also specifically examines context-specific protective processes. Indeed, all forms of stress or challenge need to be considered in terms of the interaction between people and their environments (Rutter, 2006). Therefore, studying culturally relevant protective processes in youth affected by an armed conflict, in conjunction with understanding relevant risk factors and processes, may provide crucial information for improving appropriate responses and interventions in the conflict zone (Fergus and Zimmerman, 2005; Betancourt and Khan, 2008). Of particular interest to many practitioners and policymakers are those factors which may be modified by intervention or policy. Indeed, a large debate continues about how best to respond to the mental health needs of war-affected children, youth, and their families (Summerfield, 2000; Stichick, 2001; Betancourt and Khan, 2008).

### Nurturing resilience with and by youth in conflict zones

Interventions in emergency settings have typically utilized helping professionals, such as mental health providers, humanitarian workers, and teachers. In particular, the role of mental health professionals is fundamental to treating trauma-related outcomes, as well as providing psychosocial care to the affected community (Layne et al., 2008). However, mental health providers are often in short supply, and face their challenges when working in war contexts, including feeling unprepared and less effective, burnout, and experiencing feelings of guilt or shame about being more oriented to their own needs than to the needs of their clients (Batten and Orsillo, 2002; Saakvitne, 2002; Eidelson et al., 2003; Seeley, 2003; Dekel and Baum, 2010; Nuttman-Shwartz, 2015). Additional and alternative models for building resilience in war settings are also needed.

Resilience is perhaps most powerfully nurtured through interpersonal relationships, during everyday moments with family, peers and community members (Hall et al., 2014; Newnham et al., 2015), especially for youth living in situations of atypically high-risk exposure (Sanders et al., 2017). Thus, mental health professionals argue for embedding psychosocial interventions for refugee children and youth, such as in the case of Ukraine, within intersubjective human encounters as part of daily life (e.g., supporting parents in their children’s mental health; Schwartz et al., 2022). The Tutor of Resilience program (Giordano et al., 2021) provides a model for psychosocial care developed by the Resilience Research Unit (Department of Psychology) of the Università Cattolica del Sacro Cuore to guide disasters and humanitarian responders such as local helping professionals (social workers, psychologists and other service providers) or local volunteers in the creation of a culturally and contextually sensitive approach to meeting the psychosocial needs of local populations. ToR can also be utilized to prepare youth to build resilience within their own communities.

For youth (e.g., older school-aged children, adolescents, and young adults), peers are especially important sources of support and influence (McDonald-Harker et al., 2021). With sufficient training and professional support, youth with shared experience may be especially effective promoters of resilience because peers more readily gain rapport and trust with one another and may also have greater access to one another during daily interactions. Although models of peers as facilitators of resilience interventions in conflict settings are scant, emerging evidence points to the importance of peer support among war-affected youth (Machel, 2000; Hepburn, 2006; Betancourt et al., 2013), and of engaging with peers in recreation and playful activities as part of healing and adapting to armed conflict (Loughry et al., 2006). Therefore, this study examines youth as resilience builders with peers in their communities, in the early phases of the 2023 Ukrainian-Russian war.

Preparing youth to boost resilience in their communities, when they, too, are experiencing adversity and trauma related to war is far from straightforward. Like mental health professionals who live and work in the same communities as the people they serve (Nuttman-Shwartz, 2015), youth are likely impacted by both primary and secondary trauma, as members of the traumatized community and as designated helpers serving that community (Saakvitne, 2002; Tosone et al., 2003; Nuttman-Shwartz, 2015). Unlike professionals, however, youth may have a limited understanding of trauma and resilience processes. It may be critical for intervention developers to first understand and strengthen resilience with the youth being trained to build resilience with others. In addition to building youths’ knowledge of resilience and skills for promoting it, training may also need to focus on strengthening youths’ resilience through protective processes in themselves and their communities during exceedingly challenging times of war in their home country. Research is needed to guide such approaches.

### The present study

Previous studies conducted in war settings have shown that recovery, sustainability and growth are highly dependent on the protective resources available to the community and the strengths that are nurtured before and during the negative event (Zautra et al., 2010; Ungar, 2011). Based on this assumption, the current study examined protective processes described by Ukrainian youth undergoing training in the Tutor of Resilience Program (ToR; Giordano et al., 2021, 2022) to promote resilience with peers in their communities during the early months of the war. The ToR Program offers a model of building resilience during emergencies that is flexible to meet the contextual, cultural, and individual needs of participants and communities. To inform future efforts to prepare youth to deliver resilience-building programs such as ToR, the primary study aim was to identify key protective processes Ukrainian youth depend on as they adapt to the conflict while also preparing to implement a resilience-building intervention. To inform future interventions most effectively, the study also explores individual differences in the resilience processes of these young people, contextualized within their experiences of the conflict.

## Method

### Design and context

This qualitative study involves an analysis of transcripts of discussion sessions that took place during a resilience-building intervention carried out between March 2022 (just after the beginning of the conflict) and January 2023. The intervention was aimed at training Ukrainian youth involved in peer support groups in the ToR (Giordano et al., 2021) to build resilience among their peers affected by the outbreak of the Russian-Ukrainian war. The ToR model is a culturally and contextually sensitive approach to psychosocial care developed by the Resilience Research Unit (Department of Psychology) of the Università Cattolica del Sacro Cuore. In this case, ToR was implemented with youth who had some form of designated role as a peer helper in their community, but it was originally designed to guide disaster and humanitarian aid responders, as well as local helping professionals (social workers, psychologists, and other service providers) to strengthen their work by providing culturally and contextually sensitive psychosocial support in ways that both mitigate risk and enable access to resilience-promoting resources (Giordano et al., 2021). ToR trains local facilitators (in this case, youth) to meet the needs of the community, utilizing principles of resilience as applied to the local context and culture. These principles guide them in selecting, adjusting, and/or tailoring the specific activities to enhance engagement in resilience processes that they can integrate into their ongoing community-based programming. The ToR has been applied in several humanitarian settings for a specific child, youth or family population experiencing adversity (Giordano et al., 2021; Giordano and Ungar, 2021).

### Participants

Participants were recruited by a local non-governmental organization (NGO) involved in psychosocial support in Ukraine. The inclusion criteria were: (1) being a Ukrainian youth, and (2) having a designated role as peer support in a program targeted to the Ukrainian young population. Participation in the ToR program was aimed at providing them the training in resilience to bring into their role as peer support. All participants signed an informed consent form before the beginning of the project, detailing that their participation in the program was voluntary and that discussions would be audio-recorded and potentially analyzed in research studies.

A total of 15 youth (all female), aged between 18 and 22 years (*M* = 20.20; *SD* = 1.39), participated in the program. All participants reported having been directly exposed to the war in some way since the outbreak of the conflict. Thirteen participants were still living in Ukraine; two had moved to another country. Among those who were still living in Ukraine, about half (*n* = 7) had been forced to leave their homes. Among this group, three had to move away from their close relatives because of war. About half of the participants (*n* = 7) were attending university at the time of the outbreak of the war, while the other half were employed in some capacity.

### Procedure

In the application of the ToR, 10 online discussion sessions were conducted and facilitated by the first author, a member of the Resilience Research Unit of the Catholic University of Milan. The sessions involved introducing the concept of resilience and understanding local interpretations, as well as how resilience differed from the concept of resistance. The nature of trauma and responses to trauma were also discussed, and participants were introduced to the theoretical framework of risk and protective processes (the ‘ingredients of resilience’; Giordano et al., 2021). Then, trainees are polled on the most relevant protective factors and processes that could support youth in dealing with local challenges and on how they had experienced them in response to the conflict so far. Self-care techniques were incorporated to prevent and/or mitigate the effects of secondary traumatic stress in youth involved in peer support. To provide simultaneous translation and to facilitate the conduct of the session, two professional bilingual English and Ukrainian-speaking interpreters actively participated in the sessions.

This study utilizes de-identified secondary data from this intervention; therefore, no Institutional Review Boards (IRB) oversight was required, per the common rule based on the Belmont Report. After the completion of the intervention, transcripts from the first three online sessions were de-identified and made available to the authors for analysis. The first three sessions were selected because the topics were aligned with the research questions for this study; youth engaged in dialog about resilience, and their own experiences of adversity and resilience in the context of Ukraine war. These sessions were conducted between March and May 2022; each lasted 120 min. Prompts from the focus groups that stimulated the discussion we analyzed are provided in Supplementary material.

### Analytic approach

We used the thematic analysis (Braun and Clarke, 2006, 2012; Clarke and Braun, 2013) to analyze the transcripts from the three sessions. This was undertaken by two authors and involved reading the transcripts to familiarize ourselves with the general content, followed by discussion to generate codes, coding of the full transcripts, and identification of early drafts of the themes presented below. We then reviewed the themes, discussing their significance and interrelationships, before determining together the list of distinct final themes. While undertaking the analysis, we were mindful that we intended to identify protective processes implicated in the resilience of the participants, and to that end the coding and themes could be influenced by our knowledge of these processes that have been identified in the broader research literature (Braun and Clarke, 2019). We addressed this by keeping initial codes at a very descriptive level (e.g., Byrne, 2022). We then produced memos of higher-level codes, some of which became themes, determined through consensus, and did this with continual reference to the specific sections of transcripts, to ensure that the emergent themes were strongly representative of the youths’ actual comments from the sessions. No specific software was involved in the analysis process.

## Results

### Aim 1: identify key protective processes Ukrainian youth depend on as they adapt to the conflict while preparing to implement a resilience-building intervention

We identified five protective processes common to Ukrainian youth participants: (a) Positive Thinking, (b) Sense of Control, (c) Emotion Awareness and Regulation, (d) Close Personal Relationships, and (e) Community Support. These are described below with quotations to illustrate. The results focus on these five high-level themes. For a list of codes within each theme, which may shed additional light on the themes in conjunction with the descriptions and examples throughout the Results, please see Supplementary material.

#### Positive thinking

Despite the distress that the conflict had caused, the participants described adopting perspectives and patterns of thinking that were positive, or that promoted positivity. For example, participants often described their belief that things would eventually work out: “*I believe that everything will be okay, finally*.” To some extent, this was based on their knowledge of their country’s history of emerging from national and regional conflicts. One participant, who experienced the conflict in 2014 in Severodonetsk, described this and suggested that “*…several years after the damaged cities will be restored; I think life will be back to normal, as it used to be before the war started.*”

Focusing on future positives provided some comfort: “*When I am thinking of some positive things in the future, I feel a relief.*” For one participant, who was living abroad, it was focusing on a positive endpoint that kept her going: “*I realized that everyday things are getting harder on me … [but] every time, I come to a conclusion that it will be a lot easier for me when I will be back home.*” These statements resonate with the idea of perseverance in the face of adversity, where a motivating factor is the belief that things will get better and therefore a tough period is something to bear and ‘get on with’ (the proverbial ‘light at the end of the tunnel’).

However, some participants indicated that optimism was not easy to maintain, with some struggling with the idea that things may never improve: *“On one hand I start dreaming, and on the other hand I say to myself that this may never come true.”*

Relatedly, some participants chose to focus on positive aspects of the impact of the conflict, where they recognized that it was strengthening them in some regard. On the one hand, it was recognized as tough and potentially traumatizing, but on the other, it gave them an “experience” that they could draw on if something similar were to arise again in the future. This kind of process was sometimes at a general level, in reflection of the conflict as a whole: “*…after everything is done, after this period is over, we are aware that we can cope with everything else. No other adversity will be a problem for us.*” But other times it was more focused:

*Currently I live away from my parents, which at the same time provokes my sadness; I want to live the way I used to live in the past, but I am learning to be separated from my parents, which is important, and if I go to study elsewhere, I will be familiar with being away from my parents: this is a very great advantage.*

So, there were reflections on hardship and adversity experienced that were a specific kind of positive reframing, where individuals see an ‘advantage’ of the experience that may aid them in the future, to make future hardships less difficult. While it would remain to be seen whether this process was occurring in the participants, it was their belief in it that was helpful to them: “*Definitely there are [transformations] going on inside all of us, and we are aware of that, and we are getting more enduring, and this is great.*”

#### Sense of control

Amidst the turbulence of disrupted lives, upheaval, and uncertainty, Ukrainian youth participants activated a wide variety of strategies to regain a sense of control. While some gained strength through establishing routines or making plans, others took control by redirecting their thinking and/or taking initiative to care for their physical and/or mental health needs. Some participants spoke of routines in general terms, “*I feel better when I have both feet on the ground, and when I manage to keep my daily routines,*” while others described how keeping a routine while displaced meant buying a new toothbrush and other necessities for daily living. One participant explained, “*Little things are very important: like a toothbrush, and it’s important for me to select the color I want for it, like blue or pink*.” For this participant, her mindful focus on these regular and simple occurrences, with the ability to make a choice, brought her some relief: “*Such small things bring joy.”* Another youth described the power of such small actions:

*This was the period in which I could not accept this, because it seemed to me that the worst thing was to leave my native city; I was crying, and I was looking for a railway station thinking that I could drop off and take a train back to Kiev. But then, we arrived in Lviv; we visited [a furniture store]: we bought rugs, the cups for a toothbrush, and I bought some towels, and some essentials for the room. And it was the first step to feeling better because I was doing something to decorate this dwelling to improve living conditions*.

Another strategy to regain a sense of control exhibited by the Ukrainian youth in this study was making plans with loved ones to mobilize during the war and putting those plans into action. For example, one participant described:

*In the initial days when the war started … I realized that I had to put myself together, that I had to complete certain tasks… I knew that I had to help my mother care for the younger siblings… I had to figure out where I would live. So, there were tasks to accomplish. And starting [from] days one and two, I always developed plans; and with the war going on I developed a plan, and now I feel fine*.

This same participant also described her plans for re-building her life months after this initial displacement: “*I have got a task over the recent 6 months, I would like to rent my place in Kiev and I am approaching that objective because now I am employed, so is my boyfriend.*”

Other participants took control amid an uncontrollable situation by keeping busy and doing simple actions that pleased them, such as watching a movie, shopping, or getting a haircut. This helped them to focus their attention on positive experiences. They explained, “*You watch a new movie, you have a new haircut, you have a new nail job done, when you buy a new curtain for your house*,” and “*The art, the things that inspire me – just thinking about these things make me feel better*.”

One participant displaced in Italy during the war mentioned that keeping busy with tasks that help build skills for re-establishing her life in a new country also helps reduce emotional alarm:

*What else helps me a lot when my life is full … like I had one Italian class, then another Italian class after the sessions – so when my brain has got to work, my brain gets distracted from all other problems and my alertness gets off.*

Nearly every youth expressed at least one type of intentional action that related to a sense of control. While some explicitly expressed intention to do so (“*I’m trying to retune, to rearrange myself to find ways to assist myself and my country being away from my country”*) others simply state what they do to take care of themselves and adapt (“*another benefit for me is good food and good sleep.*”). Several noted the importance of seeking professional counseling, “*I have a therapist who can listen to me and support me; Personally, I am really far away from my home place, and I was not willing to leave. So, working with a psychologist helps me to continue my development*.”

#### Emotional awareness and regulation

Nearly all the Ukrainian youth participants described the difficult emotions that they were experiencing amid the turmoil of the ongoing war with Russia. This demonstrates awareness of their own emotions (e.g., fear, uncertainty, being on alert, anxiety, worry, mistrust, feeling frozen), and often includes reflecting on the sources of their emotions, such as not knowing what will happen next, lack of control, the danger of war, separation from loved ones, and economic impacts. For example, one youth linked lack of control with her anxiety, “*I do not feel that I am controlling my own life: I cannot do anything about that and that’s why I am very anxious*.” Others reflected on the sources of their fears, such as, “*Fear of not achieving anything in life because I do not have resources to invest in my development*,” and “*Fear number two is that we will have a new reality once and forever. I am back in my native town … but there are a few people in the streets, the public transportation is not good … the fear is still there.*”

The ability to name one’s own emotions and reflect upon them is an important protective process that promotes coping with adversity and/or trauma, and healing from it (Izard et al., 2001; Katz et al., 2007). Although few youths explicitly named emotion awareness as something that helped them get through hard times, almost all exhibited such emotional awareness and/or reflection during the sessions that we analyzed, as exemplified in the preceding samples of text.

Two of the youth also explicitly noted that processing or releasing their emotions was an important coping strategy. For example, one participant described, “*It is what I am proud of: trying not to block, not to inhibit my emotions; I release my emotions and I feel better*.” Another, “*What helped me not to freeze the emotions is to give each emotion a name, and to discharge those emotions*.” A couple of the youth also reflected on how their fears negatively impacted them, “*I noticed that I am afraid of people,*” or others*, “I was hard on the secretary,”* which is a step toward positive change.

#### Close personal relationships

Participants frequently identified close interpersonal connection and supportive personal relationships as foundational to their resilience. All except one of the participants mentioned the importance of their family and/or friends. Although a few described these supportive experiences, most participants simply mentioned that staying in touch and/or talking with family and friends was essential. One youth explained, “*I also communicate with my friends and relatives, and that’s a big benefit for me, that this communication saturates me*,” and, “*Speaking about the resources, the source number one is my family*.” In addition to drawing support from loved ones, participants also noted reciprocity in their close relationships, “*I support my relative and they support me*,” and “*talking to my mother, because partially I realized that I have to keep her afloat and cheer her up, but sometimes it’s the other way around and she cheers me up*.”

In this context of war and displacement, the issue of physical proximity to loved ones was important. Some lamented being separated from friends and/or family, noting both the importance of these relationships for their resilience and the challenges in not having access to them: “*My friends who are too far away from me (and I am far away from them)*,” and “*Currently I live away from my parents, which at the same time provokes my sadness; I want to live the way I used to live in the past.”* In contrast, one participant highlighted her great fortune of being able to re-establish proximity with loved ones in a new place:

*It is nice that all my relatives are here, in Kiev, and the distance is not far – 5-min by walking distance – between my parents and my grandmothers; I live a little bit further away, but I can reach everyone just like it was in [my hometown] … we feel happy for being all together*.

The salience of close interpersonal connections was also illustrated by youth who were separated from friends and family reflecting that they found ways to establish new connections and relationships. For example, one youth mentioned, “*Once I moved to Kiev I found people to stay in touch with, I installed relationships, everything is okay*.” Another described, “*I am not home, but it’s good that during the working time, I am with distant relatives, and these distant relatives I call people that I enjoy being with; we have lots of fun*.” Another noted the power of even simple human connection during the upheaval, “*I stayed with other people, we communicated, and I think this was the second step to resilience*.”

#### Community support

Many of the youth also described broader community support that was important to their resilience. Some centered around support from the local community, such as teachers listening to children (“*they try to support the children, they try to listen to the children a little bit more, asking them what they have got in their mind,*”) tangible help in the form of transportation (“*they took me to Lviv and we were going there by car”*), and working in the community with others who are also going through similar challenges (“*staying in touch with non-resourced people … people who, just like me, they work and they are aware of different problems … so we try to figure everything out”*). Yet displacement to a country with a different language and culture also imposed challenges to community connections, as described by one participant who struggled deeply but ultimately began to gain strength through learning new languages to connect with others:

*Personally, I am really far away from my home … their pace of life is different; the attitude of people is different … I even did not want to communicate with other people because I am afraid of the need to think in English, to speak in English, and so on. Then I started attending Italian courses …now I understand some words in Italian; when we communicate with them in English, from time to time I understand their Italian words… So, it’s positive for me*.

Other mentions of community took the form of global support for Ukraine, such as solidarity through displaying flags (“*I visited some meetings in Italy, and I saw people with the Ukrainian flags, and I believe that I will be a resilient person because I see that my culture is being respected*”) and tangible support through weapons: (“*This is a type of protection: to be looking at how Ukraine is being supported … and how other countries share their weapons with us*.”) Another youth named social media as a supportive community connection, “*checking TikTok, which is a type of social network; when you realize that other people have your same type of problem: you are not alone*.”

### Results for aim 2: explore individual differences in the resilience processes of Ukrainian youth, contextualized within their particular experiences of the conflict

Despite the obvious presence of commonality in protective processes among the participants, youth also exhibited important individual differences. As described in the Method section, youth experienced the Ukrainian-Russian war in various ways, such as whether or not and to where they were displaced. In the descriptions of the five protective processes identified in results for Aim 1, we noted variations in how each protective process manifested for various youth (e.g., Sense of Control involved a range of specific strategies from re-establishing daily routines to making plans to taking care of physical and/or mental health). Yet findings also pointed to differences between individuals in which of the five protective processes applied to them, and how various protective processes come together within an individual. In this section, we call attention to such differences by highlighting three individuals with varying experiences. To protect their identities, we refer to each youth with a letter that does not correspond to their name, and provide only short examples.

Youth A’s comments in the sessions demonstrated four of the five protective processes: Positive Thinking, Sense of Control, Emotional Awareness and Regulation, and Close Personal Relationships. She talked openly about feeling fearful and mistrusting of people, and uncertainty about the future, but she also frequently used positive thinking (“it will be ok”) and explained that she grew up being explicitly taught to use positive reframing, which she found helpful. She repeatedly described the importance of making plans both in terms of preparing ahead for the war and in rebuilding her life thereafter. Youth A reflected that she was fortunate to be surrounded by family. When asked to share three words about resilience that resonate with her, she named flexibility, resolving, and recovery:

*I think that resilience is about certain levels of flexibility of the human being … finding [their] way out of difficult situations … then resolving, because when we get adjusted to something when we understand what is going on, we find the solution … finding the way to release or vent those emotions and let them go … My third word is recovery … we can recover ourselves; we can bring us back on track. And we can feel fine as we used to feel before the adversity started. But now we have that experience that we have accumulated over the adversity, and we have that resilience developed*.

Youth E also utilized four of the five protective processes, but her list included Community Support rather than Positive Thinking. In contrast to Youth A’s frequent use of Positive Thinking, Youth E actively rejected the technique of positive reframing in particular. Youth E acknowledged multiple fears (e.g., of an unknown new reality, of lost income, or inability to control) and realized that these fears led to her being “frozen” and being unkind to others. She talked of emotions being healthy and that we need to accept them, that trauma stays with us and, therefore, we have to learn to cope with it, yet also that all humans have the chance to recover. She talked of recovery in the sense of actively creating healing rather than utilizing positive thinking strategies. She reflected quite a bit about honoring individual perspectives and choices but also on the need to accept situations (e.g., that her home will not be the same when she returns) and others’ choices (parents’ decisions to leave her hometown; others’ decisions to go fight, etc.) to cope with adversity. She described the importance of establishing daily routines (buying essentials), and of connecting and staying with people, even in the absence of family, or talking with her mother on the phone. Instead of selecting three words, Youth E shared a phrase:

*I used a phrase: toughness invested in wool. This means that a person can be in different states, and we should be able to select whether to be stronger, whether to be softer, whether to allow ourselves to become softer, or to accept what is going on. So, it’s a choice between being soft and accepting the situation and being stronger, or rather being hard, and resisting the situation. This is about our different perceptions of the situations*.

In contrast to Youths A and E, Youth O exhibited just two of the five promotive processes: Emotional Awareness and Regulation, and Sense of Control. Notably, she did not describe examples of either Close Personal Relationships or Community Support. She explained that she had been away from Ukraine for 3 months and that being away continued to feel harder over time. She longed to return home but surmised that home would no longer be what it had been upon her eventual return. She talked of actively trying to find ways to cultivate her resilience, such as by working with a therapist. She explained the importance of accepting one’s situation to move on and overcome adversity. When it was her turn to name three words for resilience from her own perspective she selected choice, accepting, overcoming, and then later came back and added a fourth: frankness. She explained:

*For me, the word is choice … some people decide to move on; some people seek help … But the first thing we do is choose what to do. Then, number two: accepting the situation. If we do not accept what we have, we will not be able to move on … And number three: overcoming … we start thinking about what [has] got to be done … to overcome, to deal, to be stronger once everything is over … one of the most essential concepts related to resilience is frankness, frankness towards yourself … [if] you have difficulties with analyzing these emotions: you are either overwhelmed, or you feel this emotional deficiency. So, that’s about frankness: you have to be frank with yourself.*

## Discussion

This study set out to identify key protective processes that Ukrainian youth depend on as they adapt to the conflict while also preparing to implement a resilience-building intervention. One overarching pattern of findings is that the protective processes described by the Ukrainian youth participants are consistent with those already documented in the resilience literature, *but they are also contextually driven and experienced* in nuanced ways that are important for strategies to build resilience in this context of war and displacement. Additionally, this study documented individual differences in the resilience processes of Ukrainian youth that were contextualized within their experiences of the conflict. Findings have important implications for developing and implementing interventions to strengthen resilience in war and conflict settings, including considerations for supporting the resilience of individuals implementing the intervention, particularly in peer-to-peer youth models.

### Protective processes common to the Ukrainian youth training to build resilience with peers

This study documented five protective processes common to the youth, ages 18–22 years who were impacted by war in Ukraine and engaged in the ToR training to build skills for supporting resilience with peers: Positive Thinking, Sense of Control, Emotion Awareness and Regulation, Close Personal Relationship and Community Support. We briefly discuss each of these processes and the importance of these findings for future efforts to strengthen resilience in youth as they also build resilience with their peers in the context of adversity.

Prior research documents the benefits of positive thinking, in contrast to persistent and intrusive negative thoughts, to resilience and psychological skills (Almeida and Ifrim, 2023). In the current study, positive thinking took various forms. Some articulated general positive thoughts, consistent with hope or optimism, which have also been documented in prior research with youth affected and/or displaced by violence (Shoshani and Slone, 2016). Prior studies with refugee youth have identified the tendency to believe, expect, or hope that things will turn out well, and/or a focus on the future to be a key protective factor for resilience (Jafari, 2015; Abraham et al., 2018) that may also support positive reframing and post-traumatic growth among young refugees (Goodman, 2004; Sleijpen et al., 2016). Some participants in the current study reframed hardships by seeing an ‘advantage’ of the experience. Prior research affirms the utility of such positive reframing (Garnefski et al., 2001; Gross, 2013) as a resilience mechanism in emergency settings (Fumaz et al., 2015; Riepenhausen et al., 2022) particularly in adolescents (Jenness et al., 2016). In a particular type of positive reframing, some youth in the current study described that the adversity they experienced was strengthening them in ways that would help them cope with future adversities as well. This is in line with the concept of ‘steeling’, articulated by Rutter (2012), in which exposure to stress can have a strengthening effect, if it does not overwhelm the individual, which has been documented, for example, in Sudanese refugees residing in Australia (Khawaja et al., 2008).

Evidence from the current study brings together these various aspects of positive thinking from prior research, illustrating that they can co-occur within and between individuals in extremely adverse circumstances like war, even in a relatively small group of youth. Acknowledging this variation in the delivery of interventions and tailoring the intervention to their emerging and unique needs will be important; for example, one participant in this study actively rejected positive reframing as a resilience-building strategy but adopted other forms of positive thinking.

The second protective process detected in this study, a sense of control, is also consistent with prior research on resilience processes and may have relevance to the context of war. Amidst the turbulence of disrupted lives, upheaval, and uncertainty, people often feel a fundamental lack of control over the basic resources on which their physical and psychological well-being depend. As Sapolsky (2004) has noted, such a lack of control over unpleasant or aversive events contributes powerfully to the perception of those events as stressful. Perceived or regained sense of control is considered a core resource that contributes to resilient outcomes in individuals exposed to war settings (Mattingly, 2015; Buddelmeyer and Powdthavee, 2016; Norouzinia et al., 2017), as they are expected to minimize the perception of threat related to war-zone stressors (Schok et al., 2010). Furthermore, when people perceive even partial control over their environments, their coping efforts are more successful (Benight et al., 1997), their distress is lower, and their physiological responses are reduced (Taylor, 2017).

Findings from the current study illustrate that this sense of control can occur in a variety of different ways, such as a mindful focus on regular and simple occurrences, establishing routines or making plans, redirecting thinking, and/or taking care of their physical and/or mental health needs, and keeping busy and doing simple actions that pleased them, such as watching a movie, shopping, or getting a haircut. For youth participating in training to promote resilience with their peers, regaining a sense of control may be imperative for their ability to cope with the ongoing war sufficiently to also support resilience with others. Future research should examine the extent to which this sense of control facilitates youths’ confidence and effectiveness in supporting resilience with others. Indeed, Lowry and Lating (2002) found that reestablishing routine helps to restore a sense of safety and psychological equilibrium and assists the recovery process in emergency settings.

Prior research also identifies the processes of emotional awareness and emotional regulation as central to healing from trauma, and facilitative of resilience more generally. Emotional awareness refers to the skills of an individual to identify, explain, and discern his or her own as well as others’ emotional experiences (Lane and Schwartz, 1987) and allows a person to use the information provided by their emotions (Lischetzke and Eid, 2017) and cope with threats (Eckland and Berenbaum, 2021). In the current study, nearly all the Ukrainian youth participants exhibited the ability to identify and reflect on their various emotions (e.g., fear, uncertainty, being on alert, anxiety, worry, mistrust) and some also demonstrated awareness of the sources of such emotions (e.g., the danger of war, separation from loved ones, and economic impacts). Prior research indicates that this type of emotional awareness may be helpful to youth in learning to utilize adaptive emotion regulation strategies when faced with intense negative emotions (Van Beveren et al., 2019). Emotional regulation is the process by which individuals respond to their own emotions and how they experience and express these emotions (Grandey, 2000). Several studies highlight the importance of emotion regulation in the development of psychological resilience among youth (Wei et al., 2016; Sætren et al., 2019), and of support for individuals impacted by trauma and Post-Traumatic Stress Disorder (PTSD), which can impair emotional regulation capacities (Kulkarni et al., 2013; Lilly and Hong Phylice Lim, 2013). It is interesting that although youth in the current study showed consistent emotional awareness, only a few explicitly mentioned emotion regulation in their reflections on resilience. It could be that these youth are utilizing emotion regulation strategies without conscious recognition of their importance to resilience; future research should explore this possibility.

Findings from the current study also extend prior research on the importance of close personal relationships to resilience processes among youth experiencing adversity. Perceived social support (Haroz et al., 2013; De Nutte et al., 2017) and relationships with family and with friends (Borwick et al., 2013) serve as a deep source of strength and protection among youth exposed to war and displacement. In this study, participants most commonly described their relationships with close family members (especially parents) as important assets to their resilience, but a variety of other close relationships were also important, especially for youth who were separated from their family of origin. Indeed, findings suggest that while critically important, family and friends were not always available as sources of support in the context of war and displacement, as also documented in prior research (Zuilkowski and Betancourt, 2014). Yet, even without proximal access to family members, most participants mentioned that simply staying in touch and/or talking with family or friends has helped them to ‘make it through’ hard times. The relevance of peer relationships as a protective factor for youth during wartime highlights the importance of reciprocity in which youth not only receive support but also have the opportunity to offer emotional and tangible support to others also experiencing and being impacted by the war. Opportunities to relate to others, share, and validate personal experiences can provide individuals with the feeling that they are not isolated in their adverse environments (Borwick et al., 2013). This appeared particularly important to study participants separated from friends and family, who managed to find ways to establish new connections and relationships and to recognize the power of simple human connection during upheaval.

Finally, in line with previous studies conducted in war settings (Atari-Khan et al., 2021), participants often described the protective presence of community support, such as teachers listening to them or tangible help in the form of transportation. Engaging in the community with others who are also going through similar challenges gave youth a sense of connection and belonging (Weine et al., 2014), and a mutual understanding as it provides them with a shared experience (Goodman, 2004). Likewise, displaced participants stated that moving to a new country with a different language and culture had been particularly challenging to community connections. Studies focused on the communities in which refugee youth resettle have shown that young refugees who experience social inclusion within the host country are more likely to manage to maintain psychological well-being and display adaptive psychosocial functioning (Marley and Mauki, 2019). The current study also adds to prior evidence that community support can take the form of global support for their country or culture, such as in citizens of countries hosting refugees displaying the Ukrainian flag, or supportive and informative social media posts.

### Individual differences in protective processes in the trained Ukrainian youth

Despite the obvious presence of commonality in protective processes among the participants, this study identified individual differences among youth in different aspects related to the protective processes activated. Some individual differences have been identified in how each protective process manifested for various youth. For example, Sense of Control involved a range of specific strategies that differed from one youth to another: someone emphasized the importance of re-establishing daily routines, others highlighted the importance of making plans in terms of preparing ahead for the war and in rebuilding her life thereafter, while others stated the importance of taking care of their own physical and/or mental health.

Further individual differences have been detected in which of the five protective processes applied to them, and how various protective processes come together within an individual. This may be due do the fact that they have experienced the Ukrainian-Russian war in various ways. For example, a youth who was displaced did not recognize Close Personal Relationships or Community Support as protective processes as she had been away from Ukraine for few months. Therefore, the way she actively tried to cultivate her resilience was by working with a therapist to strengthen her inner strengths such as Emotional Awareness and Regulation, and Sense of Control. Instead, most of the youths who remained in the country recognized Close Personal Relationships and/or Community Support as fundamental protective processes to deal with the conflict situation. Furthermore, while positive reframing had been frequently mentioned by participants as relevant protective process, it turned out not be appropriate for youths who were in acute stages of adversity.

These types of specific person-to-person differences have not typically been documented in prior studies even though the literature acknowledges the variation in people’s responses to all kinds of environmental hazard (Rutter, 2006). In line with this, scholars identified the individualized principle of psychological resilience intervention which refers to the importance or providing individualized intervention programs for different individual needs and characteristic (Masten and Tellegen, 2012; Qingxing, 2023). Many influences are context-specific, making it mandatory to consider individual circumstances more closely (Rutter, 2013) and consider adaptation to adversity in terms of an interaction between individuals and their environments (Rutter, 2006). Further research is needed to improve understanding of the processes underlying individual differences in response to environmental hazards.

## Implications

Findings from this study have the following implications for interventions to promote resilience, particularly in the context of war, conflict, and displacement:

- When resilience, as a concept, is invoked to inform interventions, it is important that the focus is on making social and physical ecologies supportive, rather than changing things about an individual. This includes leveraging existing supports to develop more comprehensive psychosocial resources in ways that fit the local context. The Ukrainian youth in this study were deeply impacted by their social environments, both in terms of the adversity they experienced and in their resilience processes for adapting to it. The social environment and psychosocial resources, if adequately trained in resilience to bring into their role as peer support, can play an essential role not only as social/interpersonal PPFPs but also as tutors of resilience to activation of the PPFPs that are more individual (e.g., access to peer can help youth to be aware of their emotions and regulate them effectively, and/or to engage in enjoyable activities that help with positive thinking).
- Youth can assume the role of tutors of resilience in their own communities, which is particularly promising in the context of war where there is a limited access to mental health and psychosocial support services. The youth in this study were eager to nurture resilience in both themselves and others, demonstrated the capacity to adapt effectively to profound adversity, and deeply engaged in reflecting upon and learning about resilience in order to support their communities. Indeed, with principles of resilience as their guide, youth themselves are likely most knowledgeable about the needs of their peers and how to address them in culturally meaningful ways (Ungar, 2011). In the ToR training, for example, youth reflected upon and learned from their personal experiences in relation to key concepts regarding resilience. Results showed that it may be particularly important for them to become more cognizant of their own emotions and emotional regulation in their own resilience processes, as they in turn encourage their peers to do so This strength-based and empowering approach, where youth genuinely feel integral to the process, could be instrumental in the resilience-building process and mental health promotion (Jarlby et al., 2018). Future research on the impact of youth-centered approaches to both their own and others’ resilience is important.
- When designing resilience-focused interventions, the first step is to contextualize how resilience is defined by the local community and manifested in everyday practices. This helps to identify culturally determined outcomes that might be associated with resilience in the target community. However, local youth may not be able to articulate things that actually supported their resilience. In this sense, the ToR program encourages reflection on key concepts that may resonate with participants, while creating and sustaining a structured learning environment, which helps generate new learning starting from participants’ personal experience. Discussing the specific meaning of resilience for youths allowed participants to refocus their thoughts on relevant protective factors and processes that might help them, and their peers, in dealing with the current adversity. Giving youth room to explore aspects of resilience that fit for them and their peers, rather than presenting them a scripted or fully structured intervention, turned out to be particularly successful, also given the centrality of individuation and identify formation to adolescent or emerging adult development (Arnett, 2000; Nice and Joseph, 2023).
- Intervention development should begin by identifying which promotive or protective processes may be most accessible, effective, and impactful given a particular context, culture, and/or timing (Ungar, 2008). Indeed, providers of psychosocial support in humanitarian settings have been repeatedly accused of instilling harm when interventions overlook culturally and contextually-specific needs of populations under stress (Bernal et al., 1995, 2003; Giordano and Ungar, 2021).
- When intervening in emergency settings, it is imperative to incorporate flexibility to meet individual differences in the community members. In line with this, the current study highlighted different ways that protective processes (e.g., positive thinking or community support) were identified by participants and resonated with them.

## Limitations

The current study relied on a translation of the language and cultural meaning of participants’ comments; translation occurred simultaneously during the ToR sessions, which were then transcribed and later utilized for secondary data analysis. This process could have resulted in lost richness and meaning. A more culturally- and linguistically responsive approach would involve native speakers implementing the intervention and conducting the analysis. This was not possible to intervene quickly during a time of war. Future intervention and research efforts should consider how to strengthen such responsivity, however, as this body of work continues to progress.

A noteworthy feature of this study was the all-female sample who volunteered to participate in the ToR training from which the secondary data originated. With conscription in force at the time of the ToR training, it was not possible to recruit male participants. Gender differences have been noted in terms of trauma responses due to war (e.g., Portnoy et al., 2018) and there have been calls for considerations of conflict-related resilience in terms of gender (e.g., Juncos and Bourbeau, 2022). Findings should be interpreted with caution given the potential selection bias and gender differences. Further research should ascertain the extent to which the processes identified in this study are relevant to Ukrainian men, or whether there are important additional protective elements that men draw on for their resilience.

A further consideration of the findings is how participants themselves identified the things that they believed were important for their resilience. While this method often provided rich insight into how and when these processes were beneficial, there may have been other elements that participants did not recognize. For instance, Dubow et al. (2012) found that self-esteem was an important moderator in the relationship between exposure to violence and post-traumatic stress in Israeli and Palestinian youth. Resilience-building initiatives may therefore benefit from considering the findings of this study in conjunction with an exploration of the relevance of previously identified protective processes.

## Conclusion

Resilience-informed interventions emphasize a strengths-based approach concentrated on enhancing protective processes, instead of reducing exposure to risk or ameliorating deficits in youth, which may not always be possible in war or conflict settings (Zimmerman, 2013). Despite profound adversity and threats to mental health, we found that Ukrainian youth preparing to support resilience in their communities actively sought and demonstrated various processes to nurture their resilience as they adapted to the conflict: positive thinking, a sense of control, emotional awareness, and regulation, close personal relationships and community support. These findings highlight a promise of a resilience-building initiative with youth as a cultural asset to supporting youth more broadly. Results have implications for developing interventions to build resilience with young people experiencing profound adversities such as war and displacement. Future studies might investigate the potential of youth as builders of resilience beyond war settings, such as to building resilience in their communities in relation to natural disasters and other collective and inter-personal trauma.

Findings highlighted the importance of supporting the resilience processes within individuals (e.g., youth) being trained to promote resilience with others, and of providing flexibility for different individual and contextual needs. This study also highlights that the resilience processes that are highly common and consistently documented in research (e.g., positive thinking, close personal relationships and community support) play out in nuanced and context-specific ways that must be understood when designing and implementing interventions. With the support of programs such as Tutor of Resilience that offer tailored yet flexible approaches, resilience-building interventions can be delivered by lay people, including youth with their peers, in Ukraine and other cultures and contexts.

## Data availability statement

The data analyzed in this study is subject to the following licenses/restrictions: the data are not publicly available. Requests to access these datasets should be directed to FG, francesca.giordano@unicatt.it.

## Ethics statement

Ethical review and approval was not required for the study on human participants in accordance with the local legislation and institutional requirements. The participants provided their written informed consent to participate.

## Author contributions

FG: Conceptualization, Investigation, Resources, Writing – original draft, Writing – review & editing. SL: Conceptualization, Formal analysis, Methodology, Supervision, Visualization, Writing – review & editing, Writing – original draft. PJ: Conceptualization, Formal analysis, Writing – review & editing, Methodology, Writing – original draft. K-AK: Conceptualization, Writing – review & editing, Writing – original draft. MG: Data curation, Writing – original draft.
