# Supplementary material for: Resilience processes among Ukrainian youth preparing to build resilience with peers during the Ukraine-Russia war
Source: Front Psychol. 2024 Feb 20;15:1331886. doi: 10.3389/fpsyg.2024.1331886 (PMC10913279; doi:10.3389/fpsyg.2024.1331886)
Supplement: Supplementary file 1 [file Table_1.docx]

Supplementary Material

Resilience Processes among Ukrainian Youth Preparing to Build Resilience with Peers during the Ukraine-Russia War

Francesca Giordano*, Shannon Lipscomb, Philip Jefferies, Kyong-Ah Kwon, Marianna Giammarchi

*** Correspondence:** Corresponding Author: [Francesca.Giordano@unicatt.it](mailto:Francesca.Giordano@unicatt.it)

**Description of the Tutor of Resilience Sessions Included in this Analysis**

In the first session, participants were provided with a dynamic conceptualization of resilience, which can be seen as distributed along a continuum reflecting the different levels of the strategies adopted when reacting to a stressor – resistance, coping strategies, adaptation, and transformation (Béné and Doyen, 2018), with resilience gradually increasing through the shift from resistance to adaptation. The conversation flowed through the groups, so that participants could connect the concept of resilience to their personal experience. Participants were asked, based on their experience and the content they had absorbed so far, whether they felt they had experienced a switch from resistance to resilience since the outbreak of the war and whether they had identified any differences between the two stages (i.e., “*Have you experienced the switch from resistance to resilience since the outbreak of the war? What are the differences you experienced in the two stages?*”). This question not only allowed the participants to get more familiar with the concept of resilience while reflecting on it and connecting it with their daily experience but also to create an atmosphere of greater intimacy through some group members' sharing of their last month’s experiences.

At the end of the first session, participants were invited to identify 3 words that they felt best represented the concept of resilience in their personal life (i.e., *Based on what you have learned in the current session and on your own experience, write down 3 words that you think best represent the concept of resilience)*. The 3 words were shared with the group, to allow participants to shape together the concept of resilience in terms that they could perceive as their own. Some participants chose to explain the reasons behind their choice of certain words, sharing some of their experiences. To conclude the session, a virtual map was created in which all the words chosen by the participants were placed, so that they could be visualized as a point of start for further discussions.

The second session started with a group discussion, about what participants had learned thus far about resilience that could be applied in the peer support groups (i.e., *What have you learned about resilience that could be applied in the peer-to-peer support that you are being trained on?*). This more academic part of the discussion was not coded because participants did not describe their own protective processes. Coding focused on the rest of the discussion in which participants engaged in “Caught in a Thunderstorm” workshop (Giordano et al., 2021). Each participant was invited to identify the main challenges in their current life and the most relevant protective factors and processes that could support them in facing those challenges. The facilitator invited the participants to divide a sheet of paper in half and to draw in the upper half of the sheet the outline of few big clouds and in the bottom part of the page a big umbrella. Then participants were invited to fill the clouds with the challenges and to fill the umbrella with things that helped them deal with these challenges. The facilitator supported participants in a discussion of what had emerged. Participants concluded the session by sharing the feelings and enrichment they felt they had gained from listening and sharing their reflections with the group. As reported in literature, the use of metaphors can facilitate individuals in expressing deep emotions when dealing with adversity (Nadeau, 2006; Neimeyer, 1999; Stanley et al., 2021). A process of personal reflection and whole group reporting ensured that consensus was reached on the most important challenges and sources of resilience, much as a Delphi process (Brown, 1968) encourages stakeholders to prioritize issues in a community, yet allowed for individual differences in perspectives.

In the last part of the session, the topic of psychological trauma was gradually introduced by the conductor to the group. In order to enable the connection between theoretical aspects and concrete experiences of the topic, as well as for the youth to share part of their experiences of their last months, a Photolanguage® activity was proposed to the group by the conductor. The Photolanguage® activity was not coded, as it was not transcribed and covered a different topic.

In the third session, the facilitator introduced the manual for the ToR program, including the resilience-building principles. However, only the first part of this session, in which participants reflected on the concept of positive reframing, was transcribed and analyzed. Thus, the analysis primarily examined participants’ comments during the first two sessions, in which they reflected on their own adversity and protective processes after learning a very high-level overview of resilience. The remainder of the session, which was not transcribed or analyzed included a discussion focused on how the youth could use the ToR principles as a guide in their own resilience processes, and in their resilience-building work with their peers. As they explored their thoughts about this work with others, the participants also continued to share reflections on their own experiences, building from the first two sessions.

**List of Themes for Common Resilience Processes, with Codes from Analysis**

| **Theme** | **Codes** |
| --- | --- |
| Positive Thinking | - Hope/optimism (and challenge of hope/optimism during the war) - Positive reframing - Adversity makes us stronger/prepares us for future |
| Sense of Control | - Taking little action steps or choices - Establishing new routines - Making and implementing plans - Distraction or redirection of thought - Taking initiative/setting intentions, or making decisions to build own resilience - Taking actions for self-care: sleep, nutrition, seeing a therapist |
| Emotional Awareness and Regulation | - Self-reflection and awareness of emotions - Naming emotions and their sources - Processes or releasing emotions |
| Supportive Relationships | - Close personal relationships (family and friends) - Physical proximity to family and friends (and challenge when they are far away due to war) |
| Community Connections | - Community support (e.g., tangible such as transportation or support in working together) - Global support for country and culture of Ukraine - Social media: community and good news about the war - Challenges of finding community when displaced in a new country |

**References**

Nadeau, J. W. (2006). Metaphorically speaking: The use of metaphors in grief therapy. *Illn. Crisis Loss* 14(3), 201-221.

Neimeyer, R. A. (1999). Narrative strategies in grief therapy. *J. Constr. Psychol.* 12(1), 65-85.

Stanley, B. L., Zanin, A. C., Avalos, B. L., Tracy, S. J., and Town, S. (2021). Collective emotion during collective trauma: A metaphor analysis of the COVID-19 pandemic. *Qual. Health Res.* 31(10), 1890-1903
